# Midterm survival and neurological outcome of mild therapeutic hypothermia after cardiac arrest in a community hospital in São Paulo, Brazil

**DOI:** 10.1186/cc14695

**Published:** 2015-09-28

**Authors:** Carlos Alberto C de Abreu Filho

**Affiliations:** 1 Hospital Municipal Dr. Moysés Deutsch, Jardim Angela, São Paulo, SP, Brazil

## Introduction

Mild therapeutic hypothermia (MTH) is a powerful therapy to improve survival and neurological outcome after cardiac arrest. It is technically simple and feasible to be implemented at the community level. Our objective is to analyze midterm survival and neurological outcome of patients submitted to MTH in a community hospital of a developing country.

## Methods

Retrospective cohort study of patients treated with MTH after cardiac arrest in a community hospital in São Paulo, Brazil. After return of spontaneous circulation (ROSC), unconscious survivors of cardiac arrest were submitted to MTH, using topical ice and cold saline infusions, in order to cool patients to 32-34°C, and to maintain the goal temperature for 24 hours. Esophageal temperature was monitored for all patients during the hypothermia and rewarming process (1°C each 8 hours). Continuous intravenous sedation analgesia was maintained for 48 hours. The Glasgow Outcome Scale (GOS) was used to analyze the neurological outcome at 30 days, and 1 year after hospital discharge; GOS >3 was considered a good neurological outcome.

## Results

From January 2012 to February 2015, 148 patients were submitted to MTH after cardiac arrest; 109 patients (73.6 %) had out-of-hospital cardiac arrest and 39 (26.4 %) had intrahospital cardiac arrest. Mean age was 35.51 ± 11.08 years, 92 patients (62.1 %) were male. The mean causes of cardiac arrests were: exogenous intoxication (52.7 %), acute myocardial infarction (37.1 %) and acute respiratory failure (10.2 %). Initial rhythm was ventricular fibrillation/pulseless tachycardia (47.9 %), asystole (29.8 %) and pulseless electrical activity (22.3 %). The mean time of cardiorespiratory resuscitation was 36.4 ± 17.6 minutes, the mean time from ROSC to initiation of MTH was 167.54 ± 59.1 minutes, and the mean time from initiation of therapeutic hypothermia to goal temperature was 151.81 ± 75.4 minutes. Mean complications during MTH were: pneumonia (38.5 %), cardiac arrhythmias (31.7 %) and coagulopathy (11.4 %). Hospital mortality (30 days) was 18.9 %; among the survivors, 72.5 % of the patients had GOS >3 at hospital discharge. One-year survival was 70.9 %, among the patients who survive more than 1 year after hospital discharge, 74.5 % had GOS >3.

## Conclusion

MTH is a safe and effective therapy to improve midterm survival and neurological outcome after cardiac arrest in a community hospital.

